# Activation and polarization of circulating monocytes in severe chronic obstructive pulmonary disease

**DOI:** 10.1186/s12890-018-0664-y

**Published:** 2018-06-15

**Authors:** William D. Cornwell, Victor Kim, Xiaoxuan Fan, Marie Elena Vega, Frederick V. Ramsey, Gerard J. Criner, Thomas J. Rogers

**Affiliations:** 1 Center for Inflammation, Translational and Clinical Lung Research, Lewis Katz School of Medicine, Temple University, Philadelphia, PA 19140 USA; 2 Department of Thoracic Medicine and Surgery, Lewis Katz School of Medicine, Temple University, Philadelphia, PA 19140 USA; 3 Temple University Flow Cytometry Facility, Lewis Katz School of Medicine, Temple University, Philadelphia, PA 19140 USA; 4 Department of Clinical Sciences, Lewis Katz School of Medicine, Temple University, Philadelphia, PA 19140 USA

**Keywords:** COPD, Systemic inflammation, Polarization, Monocyte activation

## Abstract

**Background:**

The ability of circulating monocytes to develop into lung macrophages and promote lung tissue damage depends upon their phenotypic pattern of differentiation and activation. Whether this phenotypic pattern varies with COPD severity is unknown. Here we characterize the activation and differentiation status of circulating monocytes in patients with moderate vs. severe COPD.

**Methods:**

Blood monocytes were isolated from normal non-smokers (14), current smokers (13), patients with moderate (9), and severe COPD (11). These cells were subjected to analysis by flow cytometry to characterize the expression of activation markers, chemoattractant receptors, and surface markers characteristic of either M1- or M2-type macrophages.

**Results:**

Patients with severe COPD had increased numbers of total circulating monocytes and non-classical patrolling monocytes, compared to normal subjects and patients with moderate COPD. In addition, while the percentage of circulating monocytes that expressed an M2-like phenotype was reduced in patients with either moderate or severe disease, the levels of expression of M2 markers on this subpopulation of monocytes in severe COPD was significantly elevated. This was particularly evident for the expression of the chemoattractant receptor CCR5.

**Conclusions:**

Blood monocytes in severe COPD patients undergo unexpected pre-differentiation that is largely characteristic of M2-macrophage polarization, leading to the emergence of an unusual M2-like monocyte population with very high levels of CCR5. These results show that circulating monocytes in patients with severe COPD possess a cellular phenotype which may permit greater mobilization to the lung, with a pre-existing bias toward a potentially destructive inflammatory phenotype.

**Electronic supplementary material:**

The online version of this article (10.1186/s12890-018-0664-y) contains supplementary material, which is available to authorized users.

## Background

Several studies have shown that the numbers of lung macrophages are increased in patients with Chronic Obstructive Pulmonary Disease (COPD), and lung macrophage numbers increase in proportion to disease severity [1–5]. It is believed that many resident macrophages in the lungs, including those macrophages in the alveolar compartment, are derived from fetal progenitors, and are self-renewing in the lung tissue [6–9]. However, more recent evidence shows that the extravasation of monocytes into the lungs initiates differentiation of these cells into new macrophages, and these differentiated cells can persist in the lung tissue for the life span of the animal [10]. These recent immigrant macrophages can mature (or polarize) into distinct macrophage sub-populations with divergent functional activities. The M1 (classically activated) phenotype produces high levels of several pro-inflammatory cytokines [11, 12], while the M2 (alternatively activated) phenotype express high levels of mannose receptors (CD206), scavenger receptors (including CD163), IL-10, and fibronectin. The M2 cells can promote tissue fibrosis, in part, due to the expression of pro-fibrotic proteins such as fibronectin [13]. It should be pointed out that these phenotypes may actually represent two maturation stages on opposite sides of a continuum of functional capabilities.

Distinct sub-populations of monocytes can be distinguished by the expression of the surface markers CD14 and CD16. The CD14 + CD16- “classical” monocytes are considered pro-inflammatory, while the CD14 + CD16+ intermediate and CD14^DIM^ CD16+ “non-classical” cells play a role in tissue repair [14, 15]. Non-classical monocytes (5–8% of blood monocytes) expand substantially in individuals following infection or other inflammatory stimuli [16–18]. The classical monocytes are selectively recruited to inflamed tissues and lymph nodes and produce high levels of the pro-inflammatory cytokines [19]. In contrast, the non-classical monocytes, interact strongly with the luminal surface of vascular endothelial cells, and patrol the endothelial cell surface to scavenge dead cells, and certain infectious agents [14].

The non-classical monocytes remain in blood vessels until they encounter inflamed tissue, where they may extravasate [14, 20], while classical monocytes transition into and out of tissues in the absence of apparent inflammation. These monocytes continuously patrol blood vessels and most tissues, until the appropriate tissue signals are present, the cells immigrate to the lungs, and the monocyte-to-macrophage program may be initiated. Previous evidence has suggested that macrophage polarization occurs only after maturation following tissue extravasation [11, 12].

The M2 macrophage phenotype is particularly significant in the setting of COPD, since these cells can promote inappropriate tissue remodeling and fibrosis, and are believed to contribute to tissue damage in COPD [21–24]. We examined the monocytes in patients with moderate and severe COPD to determine whether these cells express markers indicative of either the M1 or M2 phenotype, and whether COPD severity varies with the pattern of phenotypic expression. We show that patients with severe COPD have unusually elevated levels of the activation marker CCR5 and M2-like markers. We propose that these populations of monocytes likely give rise to disease-promoting lung macrophages in severe COPD.

## Methods

### Subject selection

Subjects with moderate to severe COPD, current smokers without airflow obstruction (healthy smokers), and healthy nonsmokers were recruited. This study was conducted in accordance with the amended Declaration of Helsinki. Institutional Review Board approval was obtained from the Temple University Institutional Review Board, protocol 20,567, and all subjects signed written informed consent. COPD subjects were selected with an FEV_1_ between 30 and 60% predicted. Healthy smokers were currently smoking, had no airflow obstruction, and had a smoking history ≥ 10 pack-years. Subjects with allergic rhinitis, acute or chronic sinusitis, upper respiratory tract infection, or COPD exacerbation ≤ 6 weeks of the screening visit were excluded. To reduce the effects of steroids, subjects receiving inhaled or oral steroids discontinued use > 4 weeks prior to enrollment. A summary of the subject demographics is presented in Table 1.

**Table 1:** Demographic data for the study subjects

	Age (years)	Gender (M/F)	Race (AA/C/H/As)	Pack-Years	FEVI_1_ (% Pred)	FEV1/FVC	Current Smoke (Y/N)
Normal	50 (2.0)	9/5	1/10/1/2	N/A	88.1 (2.9)	81.4 (4.1)	0/14
Smoker	49.6 (1.5)	4/9	11/2/0/0	26.4 (3.1)	101.9 (4.6)	96.4 (1.8)	13/0
COPD – M	59.9 (3.9)	5/4	8/1/0/0	29.6 (7.9)	55.1 (1.6)	56.6 (3.7)	6/3
COPD – S	62.3 (2.3)	10/1	10/1/0/0	39.9 (5.6)	36.6 (1.7)	38.6 (3.1)	2/11

FEV_1_ = Forced Expiratory Volume in 1 s

FVC = Forced Vital Capacity

### Isolation of PBMCs

Venous blood was collected into vacutainers containing EDTA. The blood was layered onto Ficoll Hypaque (GE Healthcare) and centrifuged to separate the PBMCs and plasma. PBMCs were collected, washed with HBSS, and stained for flow cytometric analysis.

### Analysis of PBMCs by flow cytometry

PBMC’s (1 million) were resuspended in FACS staining buffer (BD Biosciences) and blocked with human IgG (Sigma; 20μg) for 30 min on ice. Cells were washed and resuspended in FACS buffer containing a combination of antibodies including CD3-V500 (BD Biosciences; clone UCHT1), CD14-QDot605 (Life Technologies; clone TüK4), CD16-V450 (BD Biosciences; clone 3G8), CD163-PE (Trillium; clone MAC2–158), CD206-APC-Cy7 (Biolegend; clone 15–2), CD25-Alexa700 (Biolegend; clone BC96), CCR2-Alexa647 (BD Biosciences; clone 48,607), CCR5-PE-Cy7 (BD Biosciences; clone 2D7/CCR5), IL13Rα1-PerCP-Cy5.5 (R&D Systems; clone 419,718), and CX3CR1-FITC (MBL International; clone 2A9–1) and incubated on ice for 30 min. Cells were washed with FACS buffer followed by centrifugation. Cells were resuspended in 2% paraformaldehyde and incubated on ice for 10 min. Cells were centrifuged and resuspended in FACS buffer for acquisition of events using and LSRII cytometer (BD Biosciences).

Cytometer Setup & Tracking, as well as mid-range (“rainbow”) beads (BD Biosciences) were used daily to calibrate the instrument. In addition, compensation adjustment for each channel was performed using single stained compensation beads (BD Biosciences). At least, 250,000 events were acquired per sample using BD FACSDIVA v6.1.3 software. Debris and dead cells were gated out using forward and side light scatter. The gating strategy for the flow cytometry is presented (Additional file 1: Fig. S1).

### Statistical analysis

Monocyte means (expressed as concentrations, percentages, or fluorescence intensity) for the normal, smoker, and moderate and severe COPD groups were compared using one-way ANOVA. To adjust for multiple comparisons, post-hoc comparisons were pre-planned and limited to three pair-wise comparisons of normal (as the control group) to smoker, to moderate COPD, and to severe COPD. Adjusted *p*-values were calculated using Dunnett’s method. For patients with COPD, relationships of patient data (i.e., spirometry or pack-years) and monocyte fluorescence intensity were assessed using univariate linear regression, where data for the moderate and severe COPD groups were aggregated and analyzed as a combined COPD group. All analyses were performed using SAS 9.4. Statistical significance was defined as *p* < 0.05.

## Results

### The numbers of classical and non-classical blood monocytes are altered in severe COPD

**Fig. 1:**
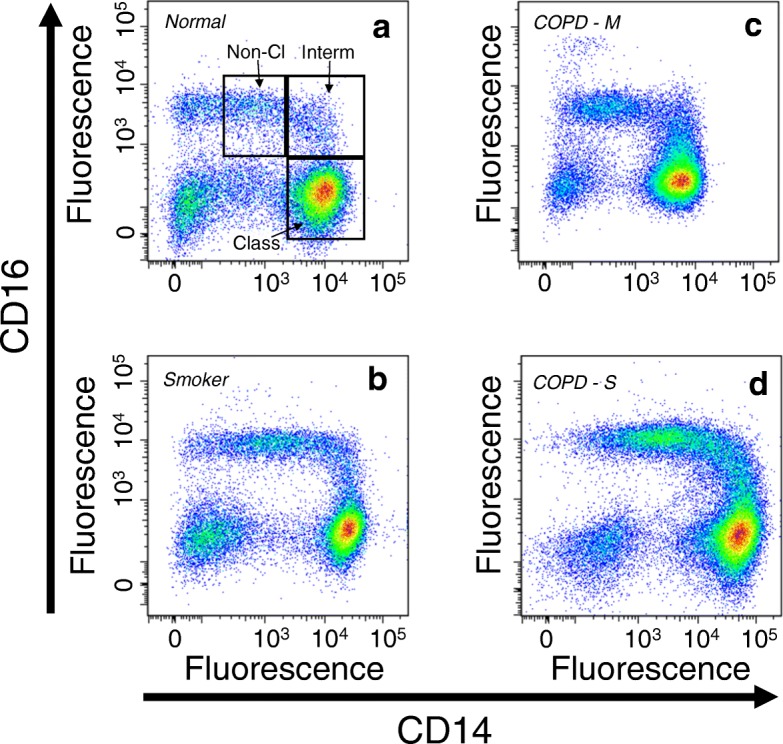
Representative flow cytometric analyses for PBMCs. Normal (**a**), smoker (**b**), moderate COPD (COPD-M) (**c**), and severe COPD (COPD-S) (**d**) were stained for expression of CD14 and CD16. Based on staining intensity, classical monocytes (CD14 + CD16-), intermediate monocytes (CD14 + CD16+), and non-classical monocytes (CD14^DIM^CD16+), as well as the total numbers of monocytes were identified

**Fig. 2:**
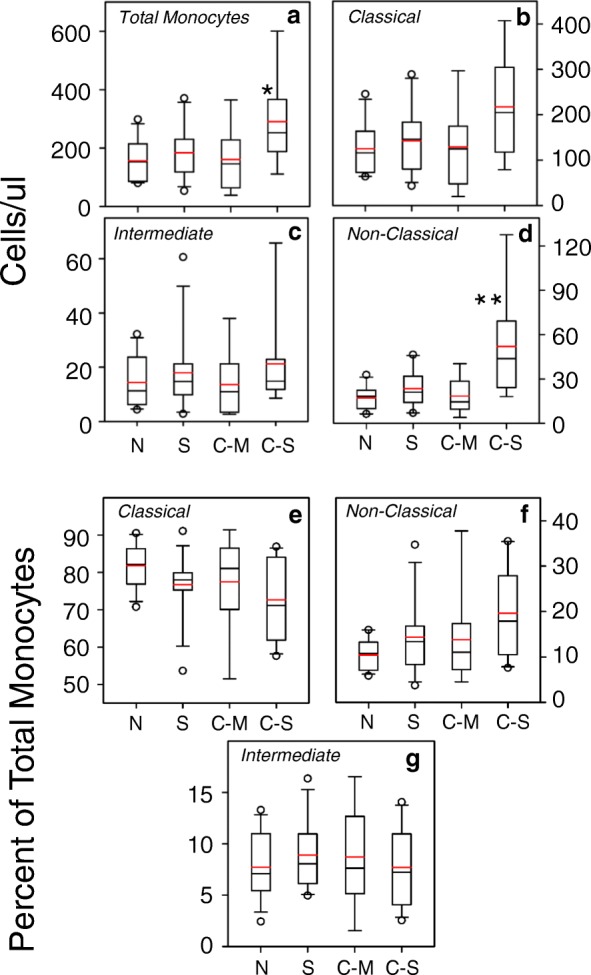
The numbers of peripheral blood monocytes are significantly increased in severe COPD patients. The total number of blood monocytes (**a**), and the numbers of classical monocytes (**b**), intermediate monocytes (**c**) and non-classical monocytes (**d**) cells are presented. The percentages of classical monocytes and non-classical monocytes are also presented. The percentages of each population were determined relative to the total number of monocytes, and data are presented for the classical (**e**), non-classical (**f**), and intermediate (**g**) monocyte sub-populations. Data are presented as box plots with the mean (red line) and median (black line). The box delineates the interquartile range, and the vertical line represents the interquartile range. * = *p* < 0.05 and ** = *p* < 0.01 relative to the normal

We used flow cytometry to evaluate the numbers of monocytes in the peripheral blood of normal subjects, current smokers without COPD, and both moderate and severe COPD. Flow cytometric analysis of the monocytes, based on CD14 and CD16 staining, demonstrates the typical pattern of classical (CD14 + 16-), intermediate (CD14 + 16+) and non-classical (CD14^DIM^16+) populations (Fig. 1). Analysis of the data (Fig. 2) show that there was a statistically significant increase in the number of total monocytes in patients with severe COPD, but no differences in the numbers of total monocytes in any other subject groups. We also determined the numbers of circulating classical, intermediate, and non-classical monocytes in each of the subject groups (Fig. 2b-d). Our results show that the numbers of each of the classical and intermediate monocyte sub-populations was modestly increased in patients with severe COPD. However, in severe COPD, a more substantial increase in non-classical monocytes was observed.

In contrast, when judged as a proportion of the total monocytes, the data show (Fig. 2e) that classical monocytes represent a modestly reduced proportion of the total monocytes in patients with severe COPD. At the same time, the proportion of non-classical monocytes (Fig. 2f) was also only modestly increased in severe COPD patients. Finally, the proportion of intermediate monocytes in these subject groups is not significantly different (Fig. 2g), and we chose to focus our further analysis on the classical and non-classical monocyte sub-populations.

### The expression of activation markers in sub-populations of monocytes in severe COPD

We evaluated the level of expression of the activation and homing proinflammatory chemokine receptors CCR2 and CCR5 in each of our subject groups. The results from the analysis (Additional File 2: Fig. S2a-d) shows modest, but statistically insignificant, changes in the expression of both of these receptors by both classical and non-classical monocytes. We also assessed the percentage of monocytes which co-express these important chemoattractant receptors, and the results show that the level of co-expression was not significantly altered in any of the subject groups, for either classical or non-classical monocytes (Additional file 2: Fig. S2e & f).

### The expression of M2 macrophage-associated markers is altered in normal smokers and patients with COPD

**Fig. 3:**
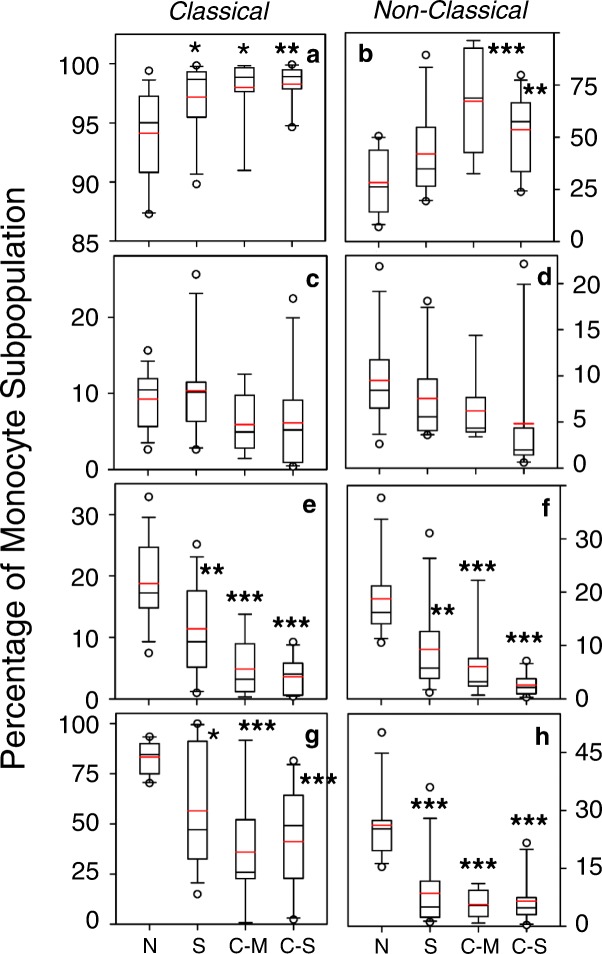
Altered composition of monocyte sub-populations in smokers and COPD patients. Classical (**a**, **c**, **e**, and **g**) and non-classical (**b**, **d**, **f**, **h**) monocytes were stained for CD163 (**a**, **b**), CD25 (**c**, **d**), CD206 (**e**, **f**), and IL-13Rα1 (**g**, **h**) expression. The data are presented as the percentage of total classical or non-classical monocytes for each group. *** = *p* < 0.001 are relative to the normal

We assessed the numbers of monocytes expressing the M2-associated markers CD163, CD206 and IL-13Rα1. We found that the percentage of both classical and non-classical monocytes expressing CD163 (Fig. 3a & b) was significantly increased in both the moderate and severe COPD groups. There was also a significant increase in CD163 expression on classical monocytes from smokers. At the same time, the percentage of cells expressing the M1-marker CD25 was not different when comparing each of the subject groups (Fig. 3c & d) with the normal controls. In contrast, the percentage of monocytes expressing either CD206 or IL-13Rα1 were reduced in both the classical and non-classical monocytes (Fig. 3e-h) in both of the COPD subject groups, as well as the smokers. Overall these results demonstrate differential expression of the M1 and M2 markers in both smokers and COPD subjects.

### The level of expression of activation markers and M2-associated markers is elevated in subjects with severe COPD

We used flow cytometry to quantitatively analyze the level of expression of each of the activation and M2 markers on monocytes. Our results show that expression (on a per cell basis) of CCR2, but not CCR5 (Additional file 3: Fig. S3a, b, d, & e), was significantly reduced on non-classical monocytes from subjects with moderate or severe COPD, or smokers. Finally, we also evaluated the level of CD14 expression, a member of the bacterial endotoxin (TLR4) receptor complex, and we find that CD14 is modestly elevated on monocytes from moderate or severe COPD subjects, but not smokers (Additional file 3: Fig. S3c & f). Interestingly, the level of expression of CX3CR1, a chemokine receptor which promotes adhesion to inflamed vascular endothelia, was also significantly elevated on non-classical monocytes in severe COPD, but not the other subject groups (Additional file 4: Fig. S4).

**Fig. 4:**
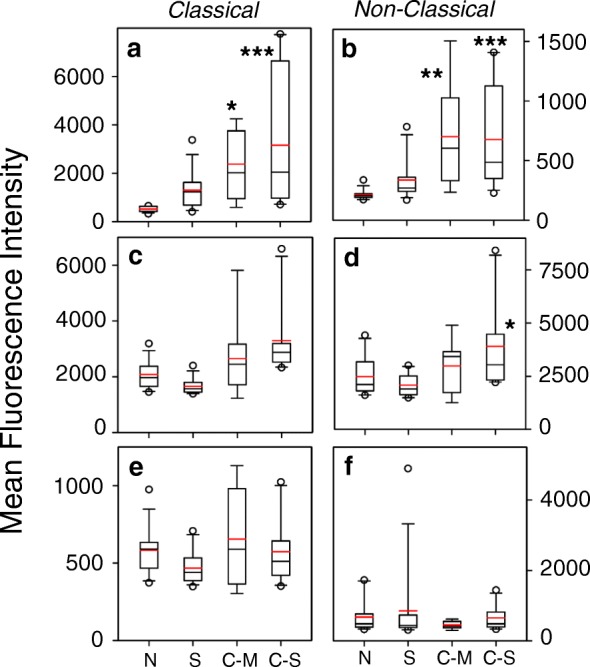
Increased CD163 and CD206 expression density in classical and non-classical monocytes in COPD patients. Classical (**a**, **c**, **e**) and non-classical (**b**, **d**, **f**) monocytes were stained for CD163 (**a**, **b**), CD206 (**c**, **d**), and CD25 (**e**, **f**) expression. The degree of expression is reported as the MFI

We also analyzed the level of expression of M1 and M2-associated markers, and our results show that the level of expression of the M2-markers CD163 and CD206 are significantly elevated on both the classical and non-classical severe COPD monocytes (Fig. 4a-d). In contrast, the level of expression of the M1-associated marker CD25 in severe COPD monocytes was not significantly different from control (Fig. 4e-f). These results show that while the proportions of cells that express CD206 are reduced in severe COPD (Fig. 3), the level of expression of both CD163 and CD206 on the cells which are positive for these markers, was substantially increased.

### Identification of a novel M2-like monocyte subset which emerges in severe COPD

**Fig. 5:**
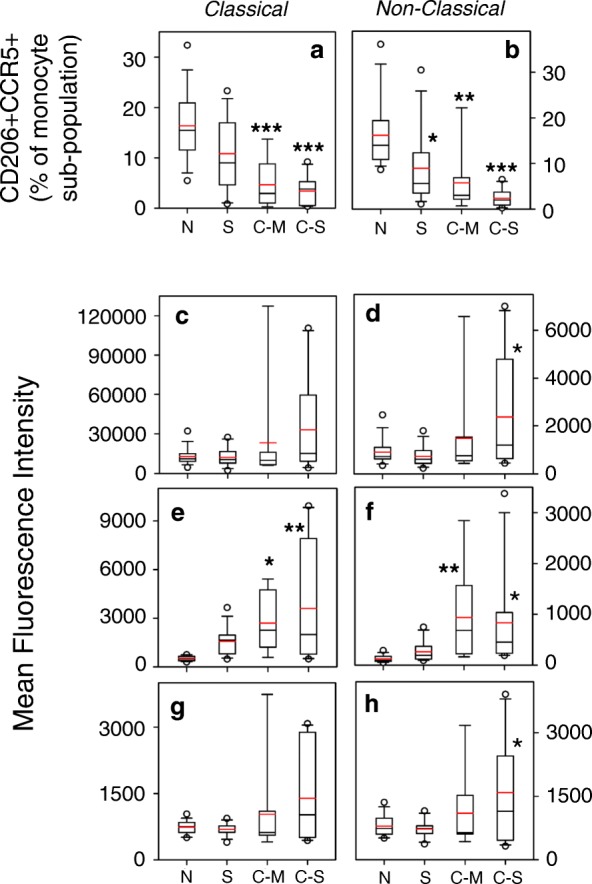
Reduced numbers of CD206 + CCR5+ monocytes with increased inflammatory phenotype in severe COPD. CD206 + CCR5+ classical (**a**, **c**, **e**, **g**) and CD206 + CCR5+ non-classical (**b**, **d**, **f**, **h**) monocytes were also stained for CD14 (**c**, **d**), CD163 (**e**, **f**) and CCR5 (**g**, **h**) expression. The degree of expression is reported as the MFI

**Fig. 6:**
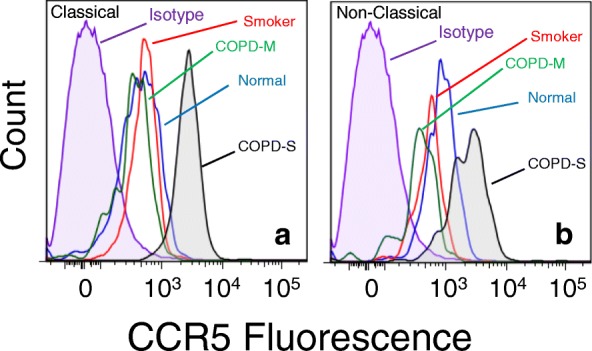
Elevated expression of CCR5 in CD206 + CCR5+ monocytes in severe COPD. Panels a and b are representative histograms of CCR5 expression on CD206 + CCR5+ classical and non-classical monocytes shown in Fig. 5. Results are representative of the 11 COPD-S patients

We attempted to determine whether the elevated level of M2-associated marker expression in monocytes from the severe COPD subjects might reflect the presence of a specific sub-population monocytes in the subjects with severe COPD. We first assessed the presence of monocytes which co-express both CD206 and CCR5 within both the classical and non-classical monocyte populations in each group. Our results (Fig. 5a, b) show that the proportion of monocytes with the CD206 + CCR5+ phenotype was reduced in severe COPD, smokers and moderate COPD patients. Moreover, when the data are expressed on the basis of cell number, the same pattern was observed (Additional file 5: Fig. S5). However, further analysis of these CD206 + CCR5+ cells shows that the level of expression of CD14 (Fig. 5c & d), the M2-marker CD163 (Fig. 5e & f), and CCR5 (Fig. 5g & h) were substantially and significantly increased in severe COPD. The levels of expression of CD163 were also significantly elevated in moderate COPD patients, but otherwise, these markers were not elevated on monocytes from smokers or moderate COPD patients. More detailed analysis of the expression of CCR5 on these CD206 + CCR5+ cells shows that the very high level of expression of CCR5 (Fig. 6) was unique and novel on the monocytes in severe COPD. These results show the emergence of monocytes with a unique high level of both CD206 and CCR5 expression, in both the classical and non-classical monocyte sub-populations.

## Discussion

The results reported here demonstrate that the number of circulating monocytes was significantly increased in patients with severe COPD, and this increase was most prominent for the non-classical monocyte population. The elevated number of circulating monocytes was not observed for smokers without COPD, or patients with moderate COPD. These results are consistent with previous studies showing that the numbers of lung macrophages are significantly elevated in patients with COPD [25–28]. A previous report has shown that the numbers of lung macrophages increases approximately 12-fold in severe COPD, and this elevation is not observed in patients with moderate COPD [28]. However, we believe the present report is the first to show that the increase in circulating monocytes in severe COPD is most significant in the non-classical population.

The non-classical sub-population functions to patrol the vasculature, in part by taking advantage of the expression of CX3CR1 [29, 30], and appears to play a significant role in clearing “damaged” endothelial cells, particularly at sites of inflammation [20, 31]. In contrast, the classical monocytes circulate in and out of normal tissues, and patrol for antigens which can be transported to lymph nodes. In inflamed tissues, these cells may also differentiate into macrophages and remain in the inflamed organ [32]. Monocytes which are recruited to the lung first reside in the parenchyma, and then under the appropriate inflammatory conditions, migrate to the alveoli [30, 33]. Indeed, the phenotype of the macrophages in the interstitium have a greater similarity to blood monocytes than to alveolar macrophages. Finally, the phenotype of monocytes which migrate into the lung is important. The non-classical monocytes when recruited to inflamed lung tissue is preferentially differentiated into the M2-type of macrophage, while the classical monocyte sub-population is a more typical source of M1-type macrophages [20]. Of course, it is important to appreciate that macrophage phenotypes are highly plastic, and environmental factors can have an effect on the functional activity of macrophages in any tissue. The M1 vs M2 paradigm should be evaluated with caution given the spectrum of phenotypes that can be derived from these cells in a given disease process [34], and the plasticity of these cells are particularly apparent in the alveolar compartment [35].

We report results here which show that the frequency of cells which express of the M2 marker CD163 (haptoglobin/hemoglobin scavenging receptor) is significantly increased for both classical and non-classical monocytes. Previous studies have shown that the expression of CD163 is significantly elevated on alveolar macrophages in patients with severe COPD [24], and recent reports show that this receptor is bound by both gram positive and negative bacteria [36, 37]. These studies suggest that bacterial binding to CD163 promotes the production of a number of cytokines and promotes lung inflammation.

In contrast with CD163, the overall percentage of cells which express the M2 marker CCR5 is not significantly altered, and the frequency of cells which express the M2 marker CD206 is actually significantly reduced among smokers and both COPD patient populations. However, we characterized the monocytes which co-express CD163, CD206 and CCR5 in an effort to assess the presence of cells with a pre-M2 phenotype. While we find that the percentage of circulating classical or non-classical monocytes which express these M2 markers is reduced in both moderate and severe COPD, we have detected the emergence of populations of classical and non-classical M2-like monocytes with an unusually high level of CCR5 expression in patients with severe, but not moderate, COPD. We hypothesize that the reduction in the percentage of these cells in the blood is due to their preferential recruitment to the inflamed lungs in these patients. The development of this population of monocytes with a pre-M2 phenotype is significant because it suggests that these cells are more likely to develop into M2 macrophages once they emigrate from the bloodstream. This would be consistent with the observation that macrophages in the lungs of COPD patients are enriched for the M2-type, and the M2 functional activity is likely to contribute to the disease process [21, 22, 24, 38]. Analysis of alveolar macrophages from COPD patients shows that expression of several M1 genes is down-regulated, while a large number of M2 genes is up-regulated [39]. Moreover, COPD alveolar macrophages have been found to exhibit impaired phagocytic activity, and in particular a reduced capacity to ingest both live and dead bacteria [40, 41], which is consistent with the reduced phagocytic activity reported for the M2-type macrophage [42].

The M2-like monocytes that we have identified in severe, but not moderate, COPD possesses unusually high levels of the chemokine receptor CCR5 and is a part of both the classical and non-classical monocyte subtype. We suggest that these cells would possess a much greater capacity to traffic to sites of inflammation, since the chemokine ligands for this receptor are typically produced at higher levels in these inflamed tissues. Experimental animal studies have shown that the severity of cigarette smoke-induced emphysema is greatly attenuated in CCR5-deficient mice [43, 44], and monocytes from patients with COPD exhibit enhanced migration in response to CCL5 [45]. Moreover, levels of CCL5 (a CCR5 agonist) are significantly increased in the lungs of patients with COPD [46].

It should be pointed out that we were unable to match the various subject groups for race or gender, and this is a limitation in our study. In addition, the subjects in our “normal” cohort exhibited lung function which was somewhat lower than might be predicted. We recruited individuals who did not exhibit apparent cardiovascular disease, diabetes, rheumatic disease, or confounding illnesses.

Finally, we were unable to assess the capacity of the novel M2-like monocytes to traffic to the lungs of patients with severe COPD. This limitation is difficult to overcome given the limits of the technology that is currently available for studying cellular traffic in humans. Nevertheless, our data show that in severe COPD, populations of M2-like monocytes develop, and these cells may preferentially migrate to the inflamed lungs of the COPD patient. This would occur because these cells possess a much greater density of CCR5, and the lung produces an elevated level of a chemokine ligand for CCR5. We suggest that once these cells are recruited to the COPD lung, they are pre-programed to further differentiate into M2-type tissue macrophages. The emergence of these pathogenic monocytes is likely to accelerate the disease progression in the lung, and thus limit the sensitivity to therapeutic intervention.

## Conclusions

Our studies reveal the emergence in severe COPD of a novel population of circulating monocytes with characteristics of the M2 lung macrophage phenotype. This monocyte phenotype was not observed in either normal subjects, smokers, or patients with moderate COPD. We suggest that cells which may be precursors of the lung M2-type of macrophage develop in the circulation, and these cells may serve as a source of these lung macrophages in severe disease.

## Additional files

Additional file 1: **Figure S1.** Flow cytometry gating strategy to identify and characterize monocyte subpopulations. PMBCs were stained as described in the Methods section and at least 250,000 events per sample were collected. Singlets (red rectangle, panel a) were gated using the forward side-scatter area (FSC-A) vs height (FSC-H). From the singlets gate, monocytes (red oval, panel b) were gated using the FSC-A vs side-scatter area (SSC-A). The monocytes were further gated using CD14 vs CD16 and are indicated by the red boxes (panel c). The classical monocytes are CD14 + CD16-; the intermediate monocytes are CD14 + CD16+; and the non-classical monocytes are CD14^DIM^CD16+. From the classical gate, cells stained for CCR2, CCR5, CD163, CD206, and IL-13Ra1 are shown (panels i-m), and from the non-classical gate, the staining for CCR2, CCR5, CD163, CD206, and IL-13Ra1 are shown in panels d-h. The red histograms indicate the isotype control for each marker. The black histograms indicated the expression of each marker. (PDF 311 kb)

Additional file 2: **Figure S2.** Analysis of CCR2 and CCR5 expression by classical and non-classical monocytes. Classical (a, c, e) and non-classical (b, d, f) monocytes were stained for CCR2 and CCR5 expression. The data are presented for the percentage of CCR2-positive (a, b), CCR5-positive (c, d), and CCR2- and CCR5-double positive (e, f) monocytes. The data are presented as the percentage of total classical or non-classical monocytes for each group. (PDF 13 kb)

Additional file 3: **Figure S3.** Altered surface expression density of monocytes in COPD patients. Classical (a-c) and non-classical monocytes (panels d-f) were stained for CCR2 (a, d), CCR5 (b, e), and CD14 (c, f) expression. The degree of expression is reported as the mean fluorescence intensity (MFI). * = *p* < 0.05 and ** = *p* < 0.01 relative to the normal. (PDF 13 kb)

Additional file 4: **Figure S4.** Increased CX3CR1 expression density in CD206 + CCR5+ non-classical monocytes in severe COPD patients. CD206 + CCR5+ co-expressing cells were stained for CX3CR1, and the mean fluorescence intensity (MFI) for each patient population was determined. Results represent the mean MFI ± SEM of all subjects in each subject group. * = *p* < 0.05. (PDF 4 kb)

Additional file 5: **Figure S5.** Reduced numbers of CD206 + CCR5+ monocytes in severe COPD. CD206 + CCR5+ classical (a) and CD206 + CCR5+ non-classical (b) monocytes data were expressed as the number of cells per μl. * = p < 0.05; ** = p < 0.01; and *** = *p* < 0.001 relative to the normal. (PDF 11 kb)

## Abbreviations

COPD: Chronic obstructive pulmonary disease
PBMC: peripheral blood mononuclear cell
