# Supplementary figures and images for: Activation and polarization of circulating monocytes in severe chronic obstructive pulmonary disease
Source: BMC Pulm Med. 2018 Jun 15;18:101. doi: 10.1186/s12890-018-0664-y (PMC6003040; doi:10.1186/s12890-018-0664-y)

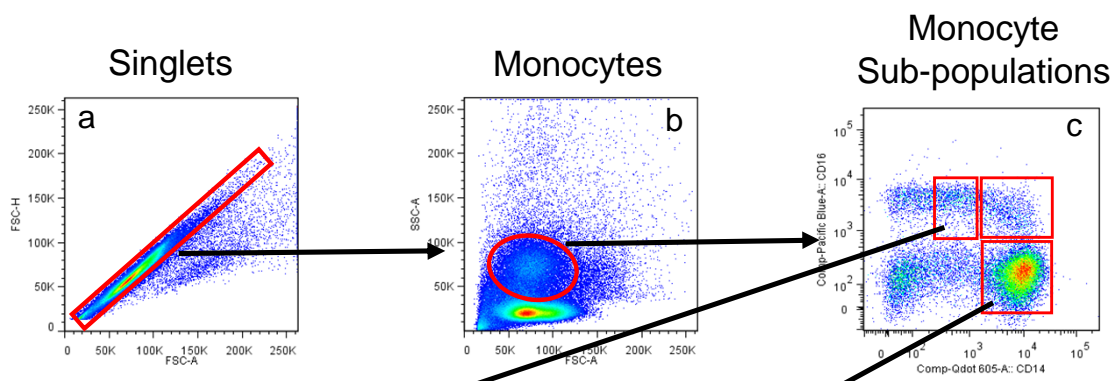

**Non-Classical**

**Classical**

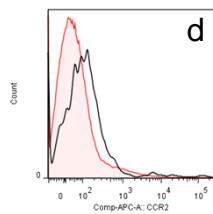

**CCR2**

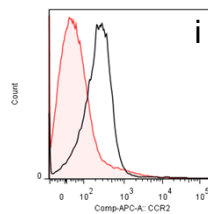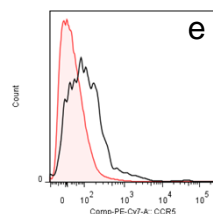

**CCR5**

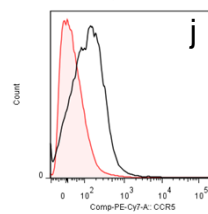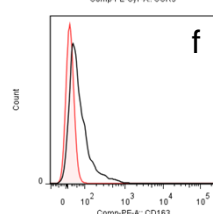

**CD163**

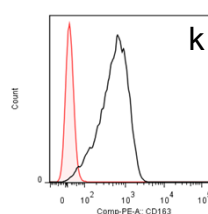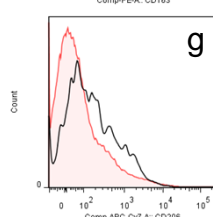

**CD206**

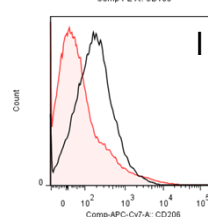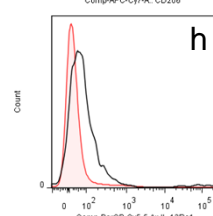

**IL-13Ra1**

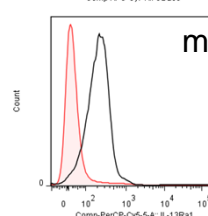

Supplement: Supplementary file 1 — Figure S1. Flow cytometry gating strategy to identify and characterize monocyte subpopulations. PMBCs were stained as described in the Methods section and at least 250,000 events per sample were collected. Singlets (red rectangle, panel a) were gated using the forward side-scatter area (FSC-A) vs height (FSC-H). From the singlets gate, monocytes (red oval, panel b) were gated using the FSC-A vs side-scatter area (SSC-A). The monocytes were further gated using CD14 vs CD16 and are indicated by the red boxes (panel c). The classical monocytes are CD14 + CD16-; the intermediate monocytes are CD14 + CD16+; and the non-classical monocytes are CD14DIMCD16+. From the classical gate, cells stained for CCR2, CCR5, CD163, CD206, and IL-13Ra1 are shown (panels i-m), and from the non-classical gate, the staining for CCR2, CCR5, CD163, CD206, and IL-13Ra1 are shown in panels d-h. The red histograms indicate the isotype control for each marker. The black histograms indicated the expression of each marker. (PDF 311 kb) [file 12890_2018_664_MOESM1_ESM.pdf]

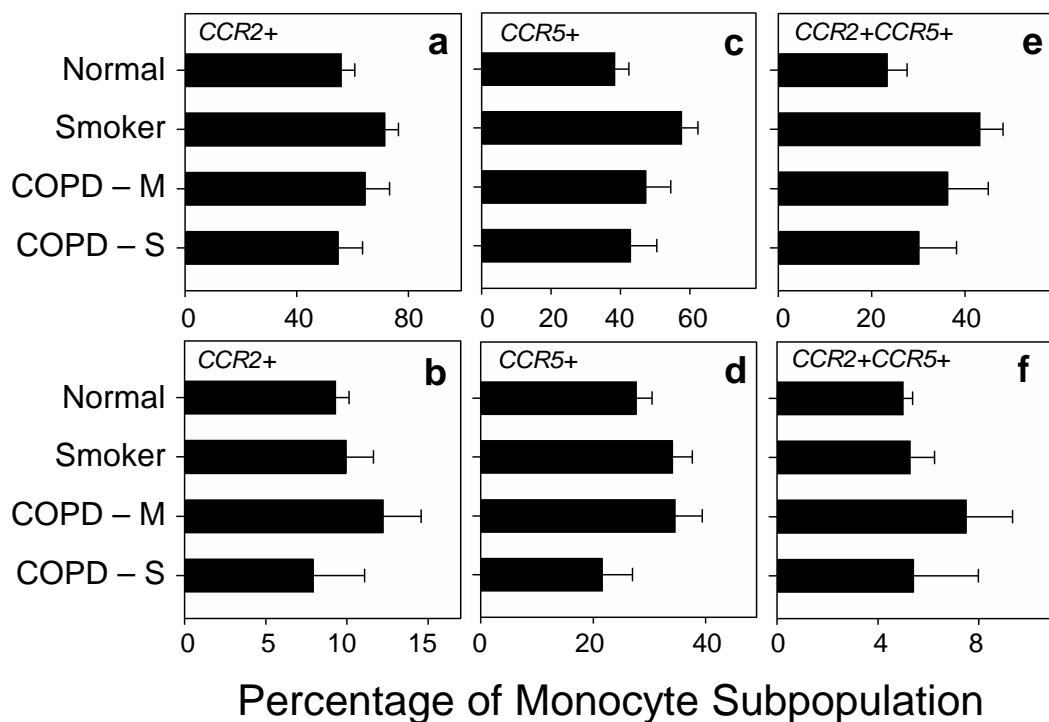

Supplement: Supplementary file 2 — Figure S2. Analysis of CCR2 and CCR5 expression by classical and non-classical monocytes. Classical (a, c, e) and non-classical (b, d, f) monocytes were stained for CCR2 and CCR5 expression. The data are presented for the percentage of CCR2-positive (a, b), CCR5-positive (c, d), and CCR2- and CCR5-double positive (e, f) monocytes. The data are presented as the percentage of total classical or non-classical monocytes for each group. (PDF 13 kb) [file 12890_2018_664_MOESM2_ESM.pdf]

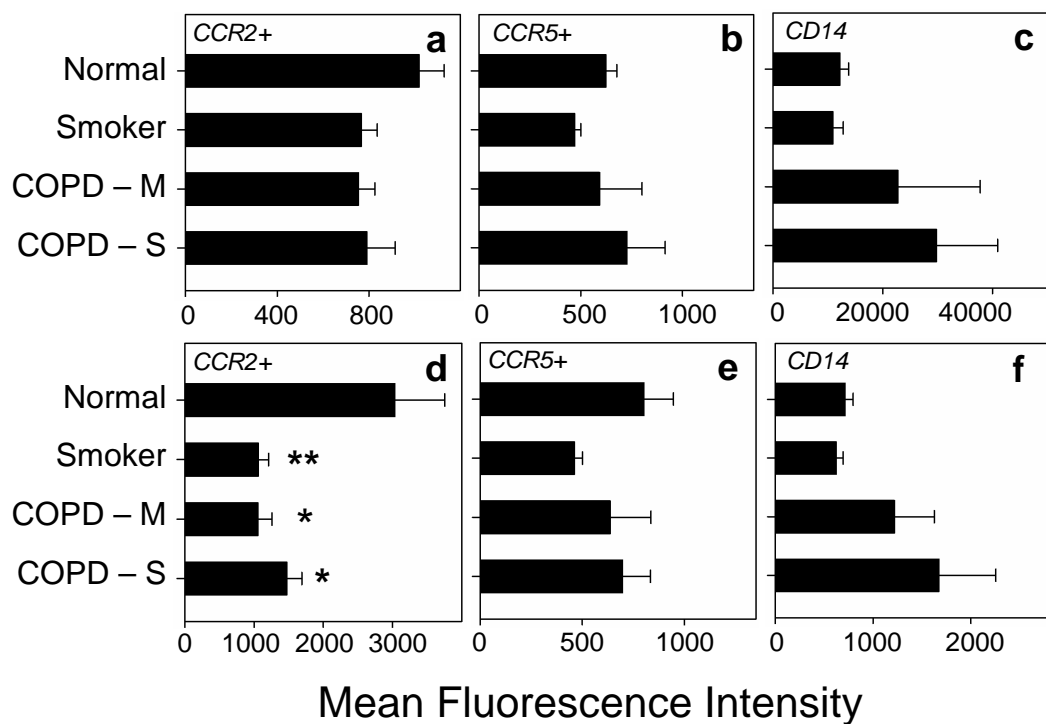

Supplement: Supplementary file 3 — Figure S3. Altered surface expression density of monocytes in COPD patients. Classical (a-c) and non-classical monocytes (panels d-f) were stained for CCR2 (a, d), CCR5 (b, e), and CD14 (c, f) expression. The degree of expression is reported as the mean fluorescence intensity (MFI). * = p < 0.05 and ** = p < 0.01 relative to the normal. (PDF 13 kb) [file 12890_2018_664_MOESM3_ESM.pdf]

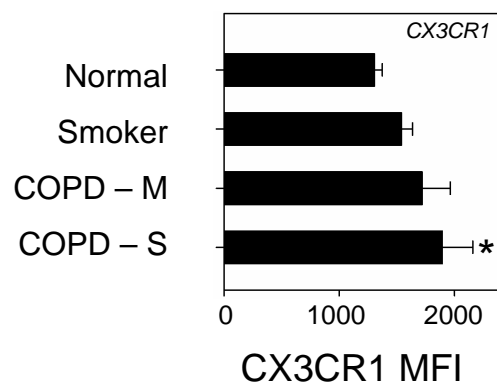

Supplement: Supplementary file 4 — Figure S4. Increased CX3CR1 expression density in CD206 + CCR5+ non-classical monocytes in severe COPD patients. CD206 + CCR5+ co-expressing cells were stained for CX3CR1, and the mean fluorescence intensity (MFI) for each patient population was determined. Results represent the mean MFI ± SEM of all subjects in each subject group. * = p < 0.05. (PDF 4 kb) [file 12890_2018_664_MOESM4_ESM.pdf]

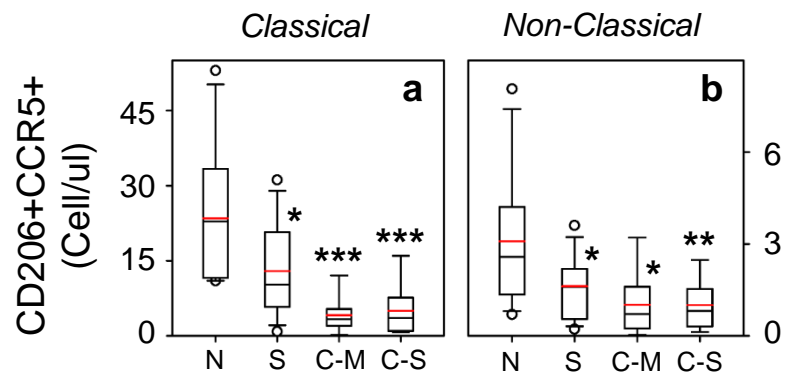

Supplement: Supplementary file 5 — Figure S5. Reduced numbers of CD206 + CCR5+ monocytes in severe COPD. CD206 + CCR5+ classical (a) and CD206 + CCR5+ non-classical (b) monocytes data were expressed as the number of cells per μl. * = p < 0.05; ** = p < 0.01; and *** = p < 0.001 relative to the normal. (PDF 11 kb) [file 12890_2018_664_MOESM5_ESM.pdf]
